# Progressive Supranuclear Palsy and Corticobasal Degeneration: Pathophysiology and Treatment Options

**DOI:** 10.1007/s11940-016-0422-5

**Published:** 2016-08-15

**Authors:** Ruth Lamb, Jonathan D. Rohrer, Andrew J. Lees, Huw R. Morris

**Affiliations:** 1 Department of Clinical Neuroscience, UCL Institute of Neurology, Queen Square, London, UK; 2 Dementia Research Centre, UCL Institute of Neurology, University College London, London, UK; 3 Department of Molecular Neuroscience, Queen Square Brain Bank for Neurological Disorders, University College London, London, UK

**Keywords:** Atypical Parkinsonism, Progressive supranuclear palsy, Steele-Richardson-Olszewski syndrome, Richardson’s syndrome, Corticobasal degeneration, Corticobasal syndrome, Movement disorders, Neurodegenerative diseases, Parkinson-plus syndromes, Treatment, Tauopathies, Tau protein

## Abstract

There are currently no disease-modifying treatments for progressive supranuclear palsy (PSP) or corticobasal degeneration (CBD), and no approved pharmacological or therapeutic treatments that are effective in controlling their symptoms. The use of most pharmacological treatment options are based on experience in other disorders or from non-randomized historical controls, case series, or expert opinion. Levodopa may provide some improvement in symptoms of Parkinsonism (specifically bradykinesia and rigidity) in PSP and CBD; however, evidence is conflicting and where present, benefits are often negligible and short lived. In fact, “poor” response to levodopa forms part of the NINDS-SPSP criteria for the diagnosis of PSP and consensus criteria for the diagnosis of CBD (Lang Mov Disord. 20 Suppl 1:S83–91, 2005; Litvan et al. Neurology. 48:119–25, 1997; Armstrong et al. Neurology. 80(5):496–503, 2013). There is some evidence that intrasalivery gland botulinum toxin is useful in managing problematic sialorrhea and that intramuscular botulinum toxin and baclofen are helpful in reducing dystonia, including blepharospasm. Benzodiazepines may also be useful in managing dystonia. Myoclonus may be managed using levetiracetam and benzodiazepines. Pharmacological agents licensed for Alzheimer’s disease (such as acetylcholinesterase inhibitors and N-Methyl-D-aspartate receptor antagonists) have been used off-label in PSP, CBD, and other tauopathies with the aim of improving cognition; however, there is limited evidence that they are effective and risk of adverse effects may outweigh benefits. The use of atypical antipsychotics for behavioural symptoms is not recommended in the elderly or those with demetia associated conditions and most antipsychotics will worsen Parkinsonism. Antidepressants may be useful for behavioral symptoms and depression but are often poorly tolerated due to adverse effects. In the absence of an effective drug treatment to target the underlying cause of CBD and PSP, management should focus on optimizing quality of life, relieving symptoms and assisting patients with their activities of daily living (ADL). Patients should be managed by a multidisciplinary team consisting of neurologists, physiotherapists (PT), occupational therapists (OT), speech and language therapists (SALT), dieticians, ophthalmologists, psychologists, and palliative care specialists.

## Introduction

Progressive supranuclear palsy (PSP) or Steele-Richardson-Olszewski syndrome is a progressive neurodegenerative disorder characterized by progressive gait disturbance (with poor balance and early falls) and a supranuclear gaze palsy (typically downward gaze)[1–4]. Other features may include axial and limb rigidity, motor eyelid disorders, pseudobulbar signs (dysarthria and dysphagia), and frontal/subcortical dementia [1–3]. Patients typically present in their 5th–7th decade [5, 6]. The mean onset age is 63 years and the mean survival is 6–9 years [2, 3, 5, 6]. The incidence of PSP is estimated to be between 1.4 and 5.3 per 100,000 [2, 3]. Neuropathologically, PSP is associated with the deposition of hyperphosphorylated tau as neurofibrillary tangles, neuropil threads, and fibrillary gliosis in the pallidum, subthalamic nucleus, red nucleus, striatum, substantia nigra, pontine tegmentum, oculomotor nucleus, medulla, and dentate nucleus [3, 7, 8].

Corticobasal degeneration (CBD) is a progressive neurodegenerative disease characterized by progressive asymmetrical rigidity and apraxia [9, 10•, 11]. Symptoms and presentation vary and patients may experience dystonia, myoclonus, tremor, alien limb phenomenon, motor speech disorders, eye movement disturbance, cortical dementia, and cortical sensory loss [10•, 12–14]. Patients typically present in their 6th or 7th decades (mean age of onset 64) and mean survival is 6 to 7 years [11, 15–17]. The incidence of CBD is estimated to be between 0.62 and 0.92 per 100,000 with a prevalence of 4.9–7.3 per 100,000 [9]. CBD is associated with accumulation of aggregates containing the four-repeat isoforms of tau [12, 13, 18]. Neuropathologically, CBD is defined by asymmetrical parietal and frontal cortical degeneration, with neurofilament protein-positive ballooned neurons and tau-positive astrocytic plaques and coiled bodies in oligodendrocytes [11]. However, the clinical syndrome of CBD is increasingly reported with other underlying pathologies such as Alzheimer’s disease (AD), frontotemporal lobar degeneration, PSP, dementia with Lewy bodies, and Creutzfeldt-Jakob disease; therefore, patients are often described as having a corticobasal syndrome (CBS) until a definitive diagnosis of CBD can be made [19].

The diagnoses of PSP and CBD are difficult due to the lack of specific biomarkers and is further complicated by the fact that many of the clinical features of PSP and CBD overlap with each other and with other neurodegenerative diseases, including multiple system atrophy (MSA) and disorders within the frontotemporal dementia spectrum (behavioral variant FTD—bvFTD and primary progressive aphasia—PPA) [12, 13, 15, 18].

Disease-modifying therapies for PSP and CBD have targeted tau pathology. Davunetide is thought to decrease tau phosphorylation by promoting microtubule stability; however, in a randomized double-blind placebo-controlled trial in 313 patients with PSP, although well tolerated, davunetide was ineffective, showing no improvement in the progressive supranuclear palsy rating scale or Schwab and England activities of daily living scale [20]. Glycogen synthase kinase-3 (GSK-3) is a kinase believed to play a role in the hyperphosphorylation of the tau protein. However, in a randomized, double-blind, placebo-controlled trial of 146 patients with PSP Tideglusib, a GSK-3 inhibitor, although well tolerated, was not shown to be clinically effective [21••]. Lithium has also been shown to regulate GSK-3; however, in a randomized, single-blind, placebo-controlled trial of 71 patients with AD, lithium was not shown to have any treatment effect on GSK-3 activity and did not support the notion that it reduced tau hyperphosphorylation [22]. Riluzole is considered neuroprotective and has been shown to block glutamatergic neurotransmission in the central nervous system and is well tolerated and prolongs survival in patients with amyotrophic lateral sclerosis; however, in a randomized, double-blind, placebo-controlled trial of patients with PSP, riluzole was not shown to improve survival in PSP and similarly did not improve survival in patients with MSA [23, 24••, 25, 26]. Lisuride was also shown to have no significant effect in the treatment of PSP [6].

In the absence of approved pharmacological treatments for PSP and CBD, management should be based on relieving symptoms and assisting patients with their activities of daily living [3, 9, 27]. Advanced care planning and non-pharmacological supportive therapies remain paramount in the management of PSP and CBD. Most patients will be trialed on L-DOPA and amantadine, although there is limited evidence for benefit some patients may experience modest improvement in Parkinsonism [15, 32, 61]. Botulinum toxin is helpful in reducing dystonia and in managing sialorrhoea and is particularly useful for eyelid dysmotility [23, 30, 31, 57, 60–63].

Advanced care planning should be addressed at the earliest possible opportunity, and ideally, when the patient is able (and legally competent) to communicate their wishes about their treatment preferences. Early input by the palliative care team is advised to explore the patients’ feelings about resuscitation status, ceilings of care, and artificial feeding using radiologically inserted gastrostomy (RIG) and percutaneous endoscopic gastrostomy (PEG). Where artificial feeding tubes are sited, decisions should me made about criteria for future withdrawal. Review by psychologists or psychiatrists may be helpful where patients exhibit challenging behaviors. Patient and caregiver education is important, and patients should be given plenty of information about relevant support groups. In some circumstances, patients may need a social worker who can provide advice about support from social services.

## Diet and lifestyle

Patients with CBD and PSP may suffer from swallowing difficulties, poor appetite, and gastrointestinal upset leading to malnutrition and weight loss. Input from speech and language therapists (SALTs) and dieticians is important to ensure patients maintain a healthy and balanced diet with sufficient calorie intake. Changes to diet and nutritional supplements may be required. Caregivers should be educated about how to optimize oral intake to ensure adequate nutrition and minimize risk of aspiration [28].

## Pharmacological treatments

### Class of drug—levodopa

#### Levodopa

**Indication**: Rigidity and bradykinesia
**Evidence in the literature**: A retrospective study of cases from the Queen Square Brain Bank (with a diagnosis of CBS or pathologically proven CBD) showed that 56 % of patients taking levodopa experienced mild-to-moderate improvement in their symptoms. Of these, 17 % developed dystonia and choreiform movements (level IV) [18, 29•].
  A retrospective study of patients with histologically proven CBD found no significant or sustained improvement from levodopa (level IV) [11]. Similarly, a retrospective study of 14 patients with CBD concluded that no patient had a dramatic response to levodopa [16].
  In a retrospective study of 147 patients with a clinical diagnosis of CBD, levodopa improved Parkinsonism in 24 % (level IV) [30].
  An observational study of 26 patients with CBD reported a mild, transient improvement with levodopa in *some* patients (level IV) [31].
  In a case series of ten patients with PSP, two experienced moderate transient improvement in symptoms [1]. In another case series of patients with PSP, levodopa moderately improved akinesia and rigidity (level V) [32].
**Standard dose**: Initially, levodopa 50 mg 3–4× daily, with a dopa decarboxylase inhibitor such as benserazide (as co-beneldopa) or carbidopa (co-careldopa) titrated slowly according to response, up to 800 mg daily in divided doses.
**Main drug interactions**: Hypertensive crisis with type A MAOIs.
  Enhancement of antihypertensive medication effect
**Side effects**: Nausea, vomiting, constipation, dystonia, choreiform movements, palpitations, postural hypotension, on/off episodes, psychosis, depression, and urinary retention [28].
**Special points**: Nausea and vomiting are common and should be treated with a peripheral dopamine receptor blocker such as domperidone 10 mg TDS.
  Levodopa should be coadministered with a dopa decarboxylase inhibitor to avoid peripheral conversion to dopamine and reduce peripheral adverse effects [28].
  Levodopa should not be stopped abruptly.

### Class of drug—dopaminergic agents

#### Amantadine

**Indication**: Bradykinesia and rigidity
**Evidence in the literature**: In a case study of two patients with PSP, amantadine 300 mg daily improved bradykinesia, rigidity, and range of voluntary lateral eye movements (level V) [32].
  In a case series of patients with CBD, amantadine 300 mg daily was given *without* improvement (level V) [9]
**Standard dose**: Initially, 100 mg daily, titrated slowly up to 400 mg daily as tolerated. Usually not recommended after 4 p.m. because of the risk of insomnia.
**Main drug interactions**: Concomitant use with tramadol, buproprion, and iohexol increases risk of seizures
**Side effects**: Insomnia and confusion are common. Also, postural hypotension, dizziness, gastrointestinal upset, dry mouth, headache, anxiety, anorexia, and livedo reticularis
**Special points**: Warn patients and caregivers of risk of impulse control disorders (excessive spending, gambling)
  This medication should not be stopped abruptly.

#### Rotigotine

**Indication**: Bradykinesia and rigidity
**Evidence in the literature**: In a study of 51 patients with atypical Parkinson’s syndrome (including CBD and PSP), transdermal rotigotine was shown to be effective and safe (reflected by an improvement in the Unified Parkinson’s Disease Rating Scale) (level IV) [22, 23].
**Standard dose**: Transdermal patch delivering 2–4 mg/24 h titrated slowly up to maximum of 16 mg/24 h
**Main drug interactions**: Antagonism of effects with concomitant use of antipsychotics, methyldopa, and metoclopramide. Use with sodium oxybate or alcohol may cause drowsiness, dizziness, and confusion.
**Side effects**: Postural hypotension, dry mouth, gastrointestinal upset, drowsiness, dizziness, excessive daytime sleepiness, dyskinesia, and headache
**Special points**: Warn patients and caregivers of risk of impulse control disorders (excessive spending, gambling)
  This medication should not be stopped abruptly.

### Class of drug—antipsychotics

The use of atypical antipsychotics in elderly patients with dementia is associated with increased mortality and is therefore NOT recommended to treat behavioral symptoms in patients with dementia in associated conditions [33]. Most anti-psychotics will worsen parkinsonism.

### Class of drug—acetylcholinesterase inhibitors

Current evidence for the use of acetylcholinesterase inhibitors for cognitive and behavioral symptoms in non Alzheimer’s disease dementia is inconclusive, and risk of adverse effects may outweigh the potential benefits in these patients. Treatment of these symptoms in CBD and PSP is based on off-label use of these medications [18]. A randomized, double-blind, placebo-controlled trial of donepezil in patients with PSP showed improvement in cognition but also reported a deterioration in ADL and mobility; donepezil was therefore NOT recommended in this group (level II) [1, 28]. Acetylcholinesterase inhibitors may also be associated with worsening of symptoms in FTD. In a study of 12 patients with FTD, donepezil was associated with worsening behavior (increased disinhibition and compulsive behavior) and showed no evidence of improvement in cognitive function or dementia severity (level III) [34, 35] . In a double-blind study of 36 patients with bvFTD and PPA, galantamine did not improve behavioral or language symptoms (level III) [35–37]. In another open-label study, rivastigmine was shown to improve behavioral symptoms and caregiver burden but did not slow cognitive decline (level III) [33, 38, 39].

### Class of drug—selective serotonin reuptake inhibitors

There are no randomized controlled trials of selective serotonin reuptake inhibitors (SSRIs) in CBD or PSP. A number of small studies of SSRIs in FTD have shown evidence of improvement in behavioral symptoms but no improvement in cognition [33, 40]. In a study of 35 patients with FTD, treatment with paroxetine improved repetitive ritualistic behaviors and anxiety in 75 % of patients (level IV) [41]. Another randomized controlled study of paroxetine in FTD showed significant improvement in behavioral symptoms (level II) [42]. An open-label trial of paroxetine in eight patients with FTD showed improvement in behavior but deterioration in cognition (level III) [40]. Similarly, in a double-blind, randomized, controlled cross-over trial of ten patients with bvFTD, paroxetine was associated with decreased accuracy of paired-associate learning, reversal learning, and delayed pattern recognition (level II) [40, 43].

#### Sertraline

**Indication**: Depression
**Evidence in the literature**: In a case study of a patient with CBD, sertraline was shown to significantly improve depression (reflected by an improvement in the geriatric depression scale) (level V) [28].
**Standard dose**: Initially 25 mg day titrated slowly to a maximum of 200 mg day
**Contraindications**: Monoamine oxidase inhibitors (MAOIs) should not be taken 2 weeks before or after treatment with this medication.
  SSRIs should not be used in poorly controlled epilepsy or patients with mania.
  Pimozide contraindicated
**Main drug interactions**: Risk of serotonin syndrome with concomitant use of St. Johns Wort, MAOIs, other SSRIs, and serotonin-norepinephrine reuptake inhibitors (SNRIs).
  SSRIs affect plasma concentration of some tricyclic antidepressants and antiepileptics.
  Increased risk of CNS toxicity with tramadol and lithium.
  Risk of arrhythmia when administered with drugs that prolong QT interval.
  Increased risk of bleeding with clopidogrel, non-steroidal anti-inflammatory drugs (NSAIDs), or warfarin
**Side effects**: Nausea, dizziness, dry mouth, anorexia, sweating, gastrointestinal disturbance, insomnia, QT prolongation
**Special points**: Caution in epilepsy, cardiac disease, diabetes mellitus, angle closure glaucoma, and bleeding disorders

#### Citalopram

**Indication**: Depression
**Evidence in the literature**: An open-label study of 15 patients with FTD showed a significant improvement in the Neuropsychiatric Inventory (NPI) and frontal behavioral inventory with citalopram (level III) [44].
**Standard dose**: Twenty-milligram once daily up to 40 mg once daily. However, in elderly, initiate at 10 mg once daily up to maximum of 20 mg
**Contraindications**: MAOIs should not be taken 2 weeks before or after treatment with this medication.
  SSRIs should not be used in poorly controlled epilepsy or patients with mania.
  Pimozide contraindicated
**Main drug interactions**: Risk of serotonin syndrome with concomitant use of St. Johns Wort, MAOIs, other SSRIs, and SNRIs.
  SSRIs affect plasma concentration of some tricyclic antidepressants and antiepileptics.
  Increased risk of CNS toxicity with tramadol and lithium.
  Risk of arrhythmia when administered with drugs that prolong QT interval.
  Increased risk of bleeding with clopidogrel, NSAIDs, or warfarin
**Side effects**: Nausea, dizziness, dry mouth, anorexia, sweating, gastrointestinal disturbance, and insomnia.
**Special points**: Caution in epilepsy, cardiac disease, diabetes mellitus, angle closure glaucoma, and bleeding disorders.

### Class of drug—tricyclic antidepressants

There are no pharmacologically approved treatments for depression in patients with CBD and PSP; however, tricyclic antidepressants (TCAs) are widely used in the empirical treatment of depression in Parkinson’s disease (PD) and have been shown to improve tremor and help improve sialorrhoea and urinary frequency [45, 46]. They have also been shown to improve behavioral symptoms in patients with FTD [40, 47–49].

#### Amitriptyline

**Indication**: Depression
**Evidence in literature**: In a retrospective study, amitriptyline improved Parkinsonism in a patient with autopsy-proven PSP (level IV) [50].
  In a retrospective study of patients with PSP, amitriptyline was beneficial in 32 % (level IV) [51].
**Standard dose**: Standard recommendations in adult patients are to initiate amitriptyline at 75 mg daily in divided doses or as a single dose at bedtime, increasing gradually as tolerated up to 150–200 mg. However, in elderly patients and those with concomitant disease, amitriptyline should be initiated at 10–25 mg at night and gradually titrate upward as tolerated.
**Contraindications**: MAOIs should not be taken 2 weeks before or after treatment with this medication.
  Pimozide contraindicated.
  Contraindicated in acute porphyria, manic phase of bipolar disorder, in the immediate recovery period following MI, and in arrhythmias.
**Main drug interactions**: Risk of serotonin syndrome with concomitant use of St. Johns Wort, MAOIs, other SSRIs, and SNRIs.
  TCAs affect plasma concentration of antiepileptics
  Increased risk of CNS toxicity with tramadol.
  Risk of arrhythmias when administered with drugs that prolong QT interval, in particular amiodarone.
  Increased risk of bleeding with clopidogrel, NSAIDs, or warfarin.
  Avoid concomitant use of nortriptyline due to risk of hypertension and arrhythmias.
**Side effects**: Dry mouth, blurred vision, headache, drowsiness, dizziness, weight gain, gastrointestinal upset, anxiety, agitation, and QT prolongation.
**Special points**: Avoid in elderly due to risk of confusion and amnesia.
  Avoid in liver disease.
  On cessation, reduce gradually over 4 weeks to avoid withdrawal symptoms.

#### Imipramine hydrochloride

**Indication**: Depression
**Evidence in the literature**: In a retrospective study of patients with PSP, imipramine was shown to be beneficial in 28 % of patients (level IV) [51].
**Standard dose**: Initially, 75 mg daily in divided dose, increased gradually up to 150–200 mg; however, in elderly, initiate at 10 mg daily and gradually titrate up to 50 mg.
**Contraindications**: MAOIs should not be taken 2 weeks before or after treatment with this medication.
  Pimozide contraindicated.
  Contraindicated in acute porphyria, manic phase of bipolar disorder, immediate recovery period following MI, and in arrhythmias.
**Main drug interactions**: Risk of serotonin syndrome with concomitant use of St. Johns Wort, MAOIs, other SSRIs, and SNRIs.
  TCAs affect plasma concentration of antiepileptics.
  Increased risk of CNS toxicity with tramadol.
  Risk of arrhythmias when administered with drugs that prolong QT interval.
  Increased risk of bleeding with clopidogrel, NSAIDs, or warfarin.
  Avoid concomitant use of nortriptyline due to risk of hypertension and arrhythmias.
**Side effects**: Dry mouth, blurred vision, fatigue, flushing, restlessness, palpitations, headache, drowsiness, dizziness, weight gain, gastrointestinal upset, anxiety, agitation, and QT prolongation.
**Special points**: Avoid in elderly due to risk of confusion and amnesia.
  Avoid in liver disease.
  On cessation, reduce gradually over 4 weeks to avoid withdrawal symptoms.

#### Clomipramine

**Indication**: Depression and obsessional states
**Evidence in the literature**: In a case series of a patients with bvFTD, clomipramine was shown to improve compulsive behaviors (level IV) [52].
**Standard dose**: Ten to 25 mg daily, increased gradually up to a maximum dose of 250 mg.
**Contraindications**: MAOIs should not be taken 2 weeks before or after treatment with this medication.
  Pimozide contraindicated.
  Contraindicated in acute porphyria, manic phase of bipolar disorder, in the immediate recovery period following MI, and in arrhythmias.
**Main drug interactions**: Risk of serotonin syndrome with concomitant use of St. Johns Wort, MAOIs, other SSRIs, and SNRIs.
  May affect plasma concentration of antiepileptics.
  Increased risk of CNS toxicity with tramadol.
  Risk of arrhythmias when administered with drugs that prolong QT interval.
  Increased risk of bleeding with clopidogrel, NSAIDs, or warfarin.
  Avoid concomitant use of nortriptyline due to risk of hypertension and arrhythmias.
**Side effects**: Dry mouth, blurred vision, headache, drowsiness, dizziness, weight gain, gastrointestinal upset, anxiety, agitation, QT prolongation, flushing, sweating, *rarely* allergic alveolitis.
**Special points**: Avoid in elderly due to risk of confusion and amnesia.
  Avoid in liver disease.
  On cessation, reduce gradually over 4 weeks to avoid withdrawal symptoms.

#### Trazodone

**Indication**: Depression and behavioral symptoms
**Evidence in the literature**: Significant improvement in depression was seen following treatment with trazodone in 20 patients with PD (level III) [53].
  A randomized, double-blind, placebo-controlled cross over trial of 26 patients with FTD demonstrated a significant decrease in the NPI and improvements in behavior following treatment with trazodone (level II) [40, 48]. These beneficial effects were sustained in an open-label extension of this study (level III) [40, 47]. In another open-label study of 14 patients with FTD, trazodone was shown to have a dose-dependent effect on behavioral symptoms [40, 49], but it did not improve cognition (level III) [39, 48].
**Standard dose**: Initially, 150 mg (100 mg in elderly) daily in divided doses (after food) or as a single dose at bedtime. Increased up to 300 mg daily.
**Contraindications**: MAOIs should not be taken 2 weeks before or after treatment with this medication.
  Pimozide contraindicated.
  Contraindicated in acute porphyria, manic phase of bipolar disorder, in the immediate recovery period following MI, and in arrhythmias.
**Main drug interactions**: Risk of serotonin syndrome with concomitant use of St. Johns Wort, MAOIs, other SSRIs, and SNRIs.
  May affect plasma concentration of antiepileptics.
  Risk of arrhythmias when administered with drugs that prolong QT interval.
**Side effects**: Hypertension, myalgia, arthralgia, hypersalivation, dry mouth, gastrointestinal upset, weight change; blurred vision, palpitations, dyspnea, QT prolongation.
**Special points**: Avoid in elderly due to risk of confusion and amnesia.
  Avoid in liver disease.
  On cessation, reduce gradually over 4 weeks to avoid withdrawal symptoms.

### Class of drug—hypnotics and anxiolytics

#### Diazepam

**Indication**: Dystonia and myoclonus
**Evidence in the literature**: In a study of 147 patients with a clinical diagnosis of CBD, 23 % reported improvement in myoclonus and 9 % improvement in dystonia following treatment with benzodiazepines; side effects of somnolence were reported in 26 % (level IV) [30].
**Standard dose**: Two to 15 mg daily in divided doses; can be increased to 60 mg daily (in divided dose) in spastic conditions.
**Contraindications**: Respiratory depression, sleep apnea, neuromuscular disorders, and myasthenia.
**Main drug interactions**: Opioids, antidepressants, antipsychotics, and antifungals.
**Side effects**: Fatigue and lethargy are common, also confusion, poor concentration, drowsiness, dizziness, hypotonia, malcoordination, headache, irritability, and memory loss.
**Special points**: Risk of rebound hypotension and tachycardia on withdrawal.

#### Clonazepam

**Indication**: Dystonia and myoclonus
**Standard dose**: Five hundred micrograms to 8 mg a day in divided doses if necessary.
**Contraindications**: Respiratory depression, sleep apnea, neuromuscular disorders, and myasthenia.
**Main drug interactions**: Opioids, antidepressants, antipsychotics, and antifungals.
**Side effects**: Fatigue and lethargy are common, also confusion, poor concentration, drowsiness, dizziness, hypotonia, malcoordination, headache, irritability, and memory loss.
**Special points**: Risk of dependence and withdrawal.

#### Zolpidem

Decreased neurotransmission of g-aminobutyric acid (GABA) in the striatum and globus pallidus is thought to contribute to the symptoms of PSP; therefore, drugs that act upon the GABAergic systems in the basal ganglia may be helpful in this condition [54].

**Indication**: Dystonia and myoclonus
**Evidence in the literature**: In a case study of a patient with PSP, zolpidem transiently improved speech, facial expressions, and fine motor skills. Less marked improvements were seen with eszopiclone, temazepam, and flurazepam [55].
  In another case study of a patient with PSP, zolpidem showed sustained improvements in motor and bulbar symptoms (reduced saliva pooling; improved speech, swallow, and bradykinesia) (level V) [56].
  In a case study of a patient with PSP, a single 10-mg dose of zolpidem improved akinesia, rigidity, dysarthria, and voluntary eye movements (level V) [54]. Similar results were observed in a double-blind, placebo-controlled cross-over study of ten patients with probable PSP, with significant improvements in motor function (lasting up to 2 h) following 5 mg zolpidem (level II) [54].
**Standard dose**: Five to 10 mg at night up to maximum of 60 mg daily
**Contraindications**: Respiratory depression, sleep apnea, neuromuscular disorders, and myasthenia
**Main drug interactions**: Opioids, antidepressants, antipsychotics, and antifungals
**Side effects**: Fatigue and lethargy are common, also confusion, poor concentration, drowsiness, dizziness, hypotonia, malcoordination, headache, irritability, memory loss, postural hypotension, and gastrointestinal upset.
**Special points**: Zolpidem is short acting and may only provide transient benefits. Avoid prolonged use.

### Class of drug—antiepileptics

#### Levetiracetam

**Indication**: Myoclonus.
**Evidence in the literature**: A case study of a 72-year-old patient with CBD reported reduction in debilitating myoclonus (level V) [57].
**Standard dose**: Initially 250 mg a day
**Main drug interactions**: Antidepressants, other antiepileptics, antimalarials, and antipsychotics.
**Side effects**: Depression is common and needs careful monitoring. Also, dizziness, drowsiness, weakness, fatigue, anxiety, and agitation.
**Special points**: Avoid abrupt withdrawal.

### Class of drug—anticholinergics/antimuscarinics

There is no recognized pharmacological treatment approved for hypersalivation and sialorrhea in CBD and PSP. Evidence is based on off-label use of medications observed in PD and patients with neurodevelopmental disabilities.

#### Atropine

**Indication**: Hypersalivation
**Evidence in the literature**: In the NICE full clinical guidance on the management of PD, sublingual 1 % atropine ophthalmic solution twice daily was suggested as an option for the management of hypersalivation.
**Standard dose**: One percent atropine ophthalmic solution administered sublingually, one drop twice daily.
**Contraindications**: Myasthenia gravis, urinary retention, gastrointestinal obstruction.
**Side effects**: Constipation, dry mouth, bradycardia, urinary urgency and retention, visual disturbance, and photophobia.
**Special points**: Caution in elderly.
  Avoid in patients with cognitive impairment, dementia, or hallucinations due to effects on cognition.

#### Glycopyrrolate

**Indication**: Hypersalivation
**Evidence in the literature**: In a randomized, double-blind, placebo-controlled cross-over trail of 23 patients with PD, oral glycopyrrolate 1 mg three times daily was found to be more effective than placebo in reducing sialorrhea (class II) [58].
  A review of treatments by the Movement Disorder Society concluded that glycopyrrolate *was efficacious* for the very short-term treatment of sialorrhea in PD, but there was insufficient evidence for the treatment of sialorrhea in PD exceeding 1 week (class V) [59]. There is no specific evidence relating to atypical parkinsonian syndromes.
**Standard dose**: 1 mg TDS orally.
**Contraindications**: Myasthenia gravis.
**Side effects**: Constipation, bradycardia, urinary urgency, retention, visual disturbance, and photophobia.
**Special points**: Caution in elderly.
  Avoid in patients with cognitive impairment, dementia, or hallucinations due to effects on cognition.

### Class of drug—ophthalmic preparations

#### Acetylcysteine

**Indication**: Dry eyes
**Standard dose**: Apply three to four times daily.
**Special points**: Do not use concomitantly with contact lenses.

#### Carbomers

**Indication**: Dry eyes
**Standard dose**: Apply three to four times daily or as required.
**Special points**: Do not use concomitantly with contact lenses.

#### Carmellose solution, hydroxyethyl cellulose, hydroxypropyl guar, hypromellose, liquid paraffin, paraffin (yellow, soft), polyvinyl alcohol, and sodium hyaluronate

**Indication**: Dry eyes
**Standard dose**: Apply as required.
**Special points**: Do not use concomitantly with contact lenses.

#### Sodium chloride 0.9 %

**Indication**: Dry eyes
**Standard dose**: Apply as required.
**Special points**: Suitable for contact lens wearers.

## Other treatments

### Botulinum toxin

**Indication**: Dystonia, including blepharospasm and sialorrhea
**Evidence in the literature**: In a study of 147 patients with clinical diagnosis of CBD, 6 out of 9 patients reported improvement in dystonia following botulinum toxin therapy (level IV) [30].
  In a case study of a patient with CBD, botulinum toxin therapy improved pain from a flexion deformity (level V) [9].
  In an observational study of 26 patients with CBD, all those treated with botulinum toxin showed symptomatic benefit (reflected by an improvement in the Unified Dystonia Rating Scale) (level III) [31].
  Botulinum toxin was also found to be beneficial in the treatment of sialorrhea in patients with parkinsonian disorders, with 65.22 % of the patients reporting a transient improvement in their symptoms (level V) [60].
  Botulinum toxin has also shown beneficial effects in patients with blepharospasm and apraxia of eyelid opening (level V) [61–63].
**Standard dose**: Botulinum toxin type A—usually 100–400 units (depending on individual preparation)
  Botulinum toxin type B—usually 5000–10,000 units divided between the most affected muscles by intramuscular injection.
**Contraindications**: Neuromuscular junction disorders.
**Side effects**: Transient weakness and discomfort at the injection site [18]. Dry mouth, dyspepsia, dysphagia, neck pain, voice changes, taste disturbance, headache, and blurred vision.
**Special points**: Side effects of dysphagia may be of greater risk when injecting botulinum into the salivary glands for management of sialorrhea in PSP [61].

## Assistive devices

**Urinary catheter**: There is no data in the literature to guide use of urinary catheters in CBD or PSP; however, they are a useful tool to manage urinary difficulties at the end of life, reducing caregiver burden and patient discomfort, improving hygiene, and reducing the risk of skin breakdown.

### Prisms

**Indication**: Gaze paresis patients should have an ophthalmology review to determine if interventions such as prisms could help correct double vision and to exclude additional pathology.
**Special points**: Patients should not wear prisms continuously.
  Patients should register as sight impaired.

## Interventional procedures

### Radiologically inserted gastroscopy and percutaneous endoscopic gastroscopy

**Evidence in the literature**: A systematic literature review found no evidence to suggest improved survival in patients with advanced dementia who underwent PEG insertion for dysphagia (level IV) [64]. There have been no studies on survival following PEG or RIG in CBD or PSP.

## Physical/speech/occupational therapy and exercise

**Physiotherapy**: Physiotherapists should be consulted to optimize strength, balance, posture, coordination, and mobility and to help prevent falls. Physiotherapists can also provide advice on walking aids, orthotics, and splints to prevent contractures.
**Evidence in the literature**: In a case study of a patient with CBD, repetition of facilitation exercises improved hand function and ADL (level V) [29•].
  A regular exercise program undertaken over 10 years in a patient with features of CBD and PSP was shown to decrease falls and maintain mobility, balance, and strength (level V) [33].
  In an observational study of 26 patients with CBS, motor and non-motor symptoms improved following a combination of physical therapy, pharmacological therapy, and (low frequency) repetitive transcranial stimulation (level V) [31].
**Occupational therapy**: Occupational therapists should assess patients to identify and minimize potential hazards, and they can assist in developing skills to promote independence: providing the necessary tools to assist the patient in their ADL.
**Speech therapy**: Speech and language therapists can provide solutions and strategies to help overcome communication and swallowing difficulties in those with dysphasia, dysarthria, and/or dysphagia. Close monitoring of dysphagia is essential to help prevent aspiration and maintain adequate oral nutrition and a healthy weight.
**Evidence in the literature**: In a prospective longitudinal study of 20 patients with PPA, regular speech and language therapy was shown to slow language decline (level III) [65].
  A study of two patients with PPA showed sustained improvement in language skills and greater confidence in communication following lexical retrieval training (level IV) [66]. Similar studies have also demonstrated positive effects following language treatment in patients with PPA (level IV) [67–69].

## Support services

Supportive therapy is the mainstay of management, and contact with support groups may be of benefit to patients and those who care for them [3].

**PSP Association**: The PSP Association is a registered charity dedicated to the support of people with PSP, CBD, and those who care for them. The PSP association provides information leaflets, telephone, and e-mail advice and hosts support groups and educational events.
  http://www.pspassociation.org.uk
**Cure PSP**: Cure PSP is an organization dedicated to furthering research into neurodegenerative diseases. It provides information and support to people with PSP, CBD, and related conditions, and those caring for them.
  http://www.psp.org
